# An overwinter protocol for detecting wolverines and other carnivores at camera traps paired with automated scent dispensers

**DOI:** 10.1002/ece3.11290

**Published:** 2024-05-02

**Authors:** Robert A. Long, Paula MacKay, Joel D. Sauder, Mike Sinclair, Keith B. Aubry, Catherine M. Raley

**Affiliations:** ^1^ Woodland Park Zoo Seattle Washington USA; ^2^ Idaho Department of Fish and Game Lewiston Idaho USA; ^3^ Microsoft Research, Microsoft Corporation Redmond Washington USA; ^4^ USDA Forest Service Pacific Northwest Research Station Olympia Washington USA

**Keywords:** camera trap, carnivores, fisher, noninvasive, scent lure, survey, winter, wolverine

## Abstract

Camera traps deployed with olfactory attractants are used to survey rare and elusive carnivores. Study areas with deep snowpack and rugged terrain present challenges and risks to field personnel, who traditionally must revisit camera stations regularly to refresh attractants. In such locations, alternative overwinter survey protocols that include a persistent attractant would improve both the safety and efficiency of camera‐trap surveys. We present a protocol for installing camera traps and automated scent dispensers on trees at above‐average maximum snow depth to eliminate the need for interim service visits and to enable standardized surveys to be conducted throughout the year. Our protocol proved to be effective at attracting and detecting numerous and repeated visits by wolverines, fishers, and other carnivores in two montane regions of the western contiguous United States. The volume, timing, and composition of liquid scent lure released by automated scent dispensers can be varied to target multiple species of interest, and the dispenser can be used in situations where bait rewards may influence the behavior of target species and/or pose human safety concerns.

## Graphical abstract

Camera traps (CTs) deployed with olfactory attractants are used to conduct surveys for rare and elusive carnivores, however, study areas with deep snowpack or rugged terrain present challenges to refresh attractants. We present a protocol for installing CTs and automated scent dispensers to enable consistent survey efforts throughout the year while eliminating the need for interim service visits. We also present two case studies using this protocol in montane regions of the western contiguous United States, and show that the method can be tailored to multiple species of interest and used in situations where bait rewards may influence the behavior of target species and/or pose human safety concerns.

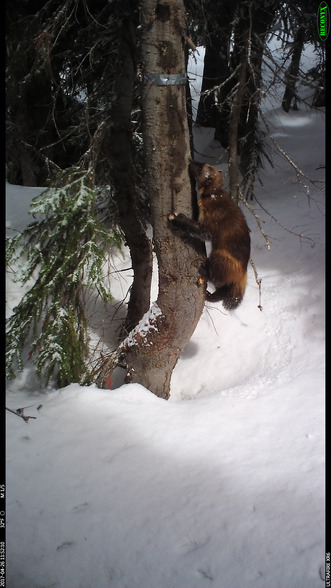

## 1 INTRODUCTION

**FIGURE 1:**
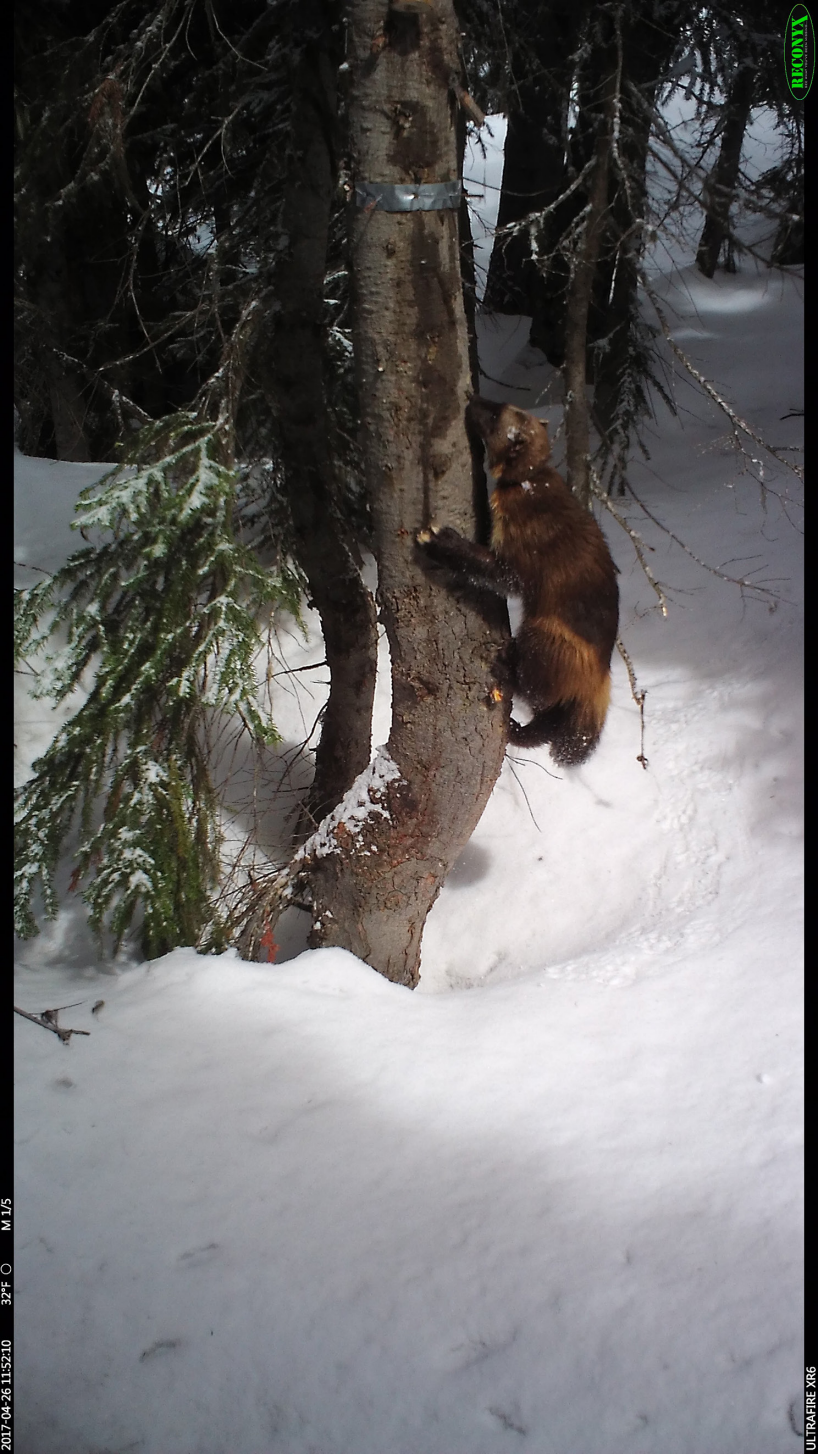
A wolverine visits a survey station in the North Cascades of Washington.

Wildlife trail cameras, typically referred to as camera traps (hereafter CTs), are an important tool for carnivore researchers (Burton et al., 2015; Kays & Slauson, 2008). CTs are noninvasive, operate over long time periods, and are capable of producing consistent detection data without animal capture or handling. Data produced from CTs deployed across large areas can yield inferences on species occupancy (Anderson et al., 2023; Lukacs et al., 2020; Rich et al., 2018), colonization/extinction (Farris et al., 2017), population temporal dynamics (Kellner et al., 2022), and animal behavior (Burton et al., 2022; Murphy et al., 2021). For species with distinctive markings (e.g., striped or spotted felids [*Felidae* spp.], wolverines [*Gulo gulo*; Figure 1]), CTs can also generate sight‐resight data for capture–recapture models to estimate population abundance and density (Anderson et al., 2023; Green et al., 2020; Rowcliffe et al., 2008).

Depending on survey objectives and target species, CTs deployed along trails or roads are typically unbaited and unscented, whereas those installed off‐trail are often paired with baits and/or scent lures. The detectability of target species may vary by approach (Barcelos et al., 2023; Holinda et al., 2020; Iannarilli et al., 2021; Mills et al., 2019). In North America, for example, canids and felids have been effectively surveyed by CTs positioned along trails and secondary roads without baits or scent lures (Anderson et al., 2023), and Holinda et al. (2020) found no effect of attractant on gray wolf (*Canis lupus*) detectability. In contrast, mustelid and ursid detection rates have generally benefitted from scent lures (Holinda et al., 2020; Iannarilli et al., 2021).

Treating CT stations with scent lures and/or baits can present a host of challenges. Lures and meat baits should be refreshed every 2–4 weeks to reliably attract carnivores, but refreshing attractants in remote locations can be hazardous and infeasible—particularly during winter. In addition, regular visits to survey sites by field personnel may disrupt normal animal movements, potentially reducing detectability even if scent lures and meat baits are replenished (Barcelos et al., 2023). When ursids are not hibernating, they are prone to consuming bait or otherwise disturbing survey stations—again, potentially decreasing the detectability of target species (R. Long, unpublished data; Zielinski et al., 2011). Meat baits can be difficult to standardize in terms of volume or odor (R. Long, unpublished data), and baits that provide wildlife with food rewards can promote food conditioning (Barcelos et al., 2023) or guarding behaviors that may increase the risk of human–carnivore conflicts (Caravaggi et al., 2020). Notably, meat baits also carry the potential risk of spreading diseases, particularly Chronic Wasting Disease (Saunders et al., 2012).

We sought to conduct CT‐based, detection‐nondetection surveys whose resulting data would enable occupancy estimation (MacKenzie et al., 2002) for wolverines in the northern Cascade Range of Washington and fishers (*Pekania pennanti*) in the Rocky Mountains of Idaho. High detection probabilities are pivotal for precise occupancy estimates, which are, in turn, essential for obtaining high power to detect population trends. Prior CT research conducted by us (R. Long, unpublished data) and others (e.g., Zielinski et al., 2015) demonstrated higher detection rates for mustelids in winter versus nonwinter. In both of our study areas, however, winter access is often restricted by rugged terrain, deep snow, high avalanche risk, relatively few roads and trails, and limitations on helicopter use due to land‐use regulations (e.g., designated Wilderness). To meet our goal, we developed a novel protocol for deploying CTs with automated scent dispensers, which (1) enables overwinter (i.e., 6–12 month) carnivore surveys without the need for interim visits by field personnel; (2) maintains consistent scent lure; (3) precludes food rewards; (4) is unaffected by snow conditions and subfreezing temperatures; and (5) detects target as well as numerous nontarget species. Here we demonstrate that our protocol is effective for detecting wolverines and fishers while also meeting the objectives described above.

Although adapted versions have now been used by other researchers (i.e., Krohner et al., 2022; Lukacs et al., 2020), this is the first time the protocol has been fully described in a scientific publication, and its advantages and challenges discussed.

## 2 MATERIALS AND METHODS

### 2.1 Camera/dispenser protocol

**FIGURE 2:**
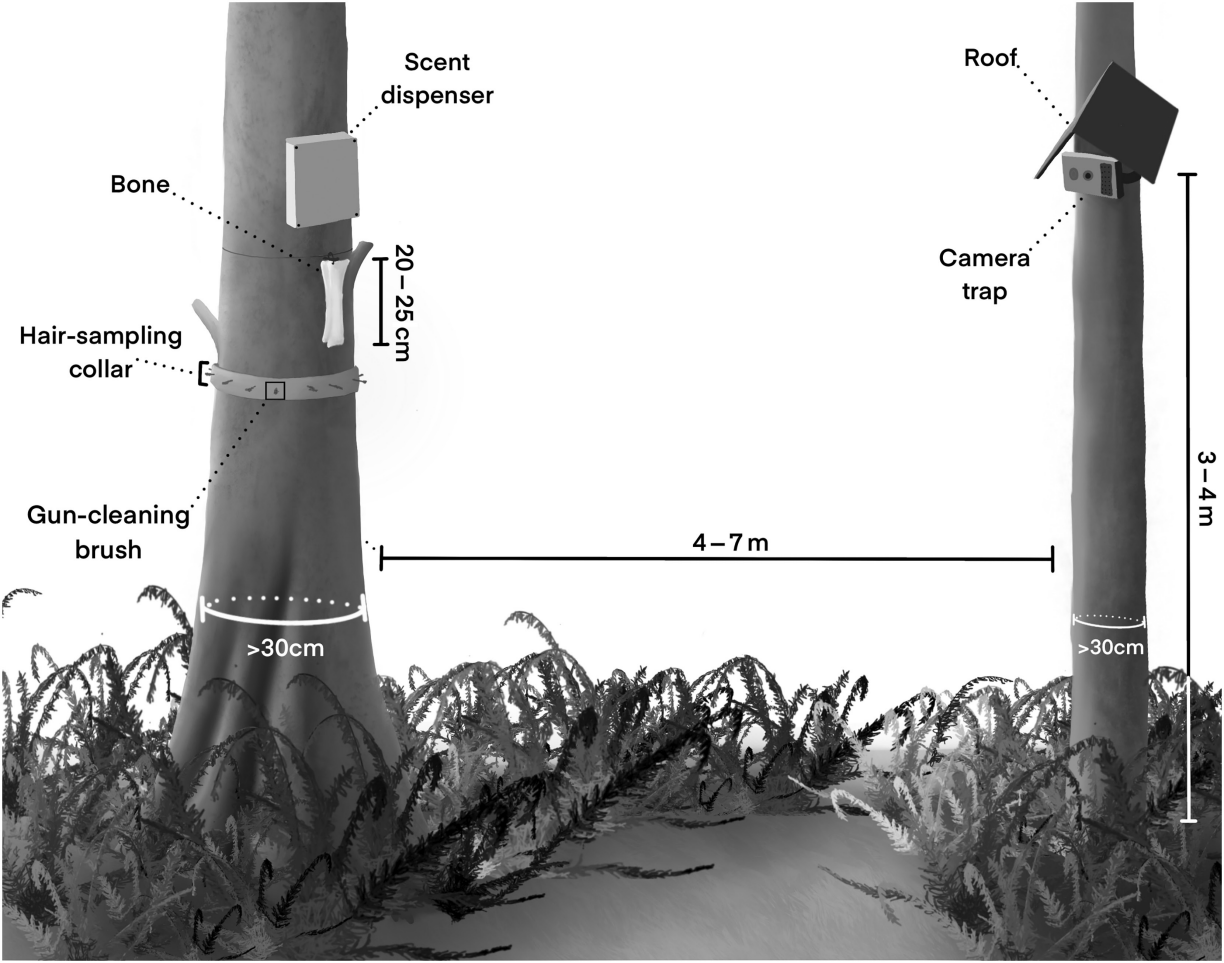
Ilustration of setup at an overwinter survey station, including a camera trap deployed vertically by rotating the unit by 90 degrees, corrugated polypropylene “roof” over the camera trap, scent dispenser, bone (hung by cable or eyebolt), and optional hair sampling brushes fastened to a corrugated polypropylene “collar”. Illustration: Samantha Kreling.

At each CT station, we installed a Reconyx‐brand CT (Holmen, WI) and an automated scent dispenser (Woodland Park Zoo, Seattle, WA) (Figure 2). The automated dispenser was invented by Long, Sauder, and Sinclair to facilitate overwinter detection‐nondetection surveys for wolverines. Powered by 8 lithium AA batteries, the scent dispenser pairs an ultra‐low voltage control board with a miniature peristaltic pump to release a pre‐selected volume of liquid at programmed intervals for >12 months, even under extreme environmental conditions (e.g., one unit was deployed in Alaska and continued to function at temperatures below −40°C [pers. comm., M. Robards; Wildlife Conservation Society]). The ingredients in our scent lure were adapted by Aubry et al. (2023) from various recipes and included liquid beaver (*Castor canadensis*) castoreum oil, pure skunk (*Mephitis* spp.) extract, anise (*Pimpinella anisum*) oil, and either commercial mustelid lure (Marten Lure, Hawbaker & Sons, Fort Loudon, PA) or fish oil. The remaining volume comprised a 60:40 mixture of food‐grade propylene glycol and water to reduce the freezing point to ~−40°C.

**FIGURE 3:**
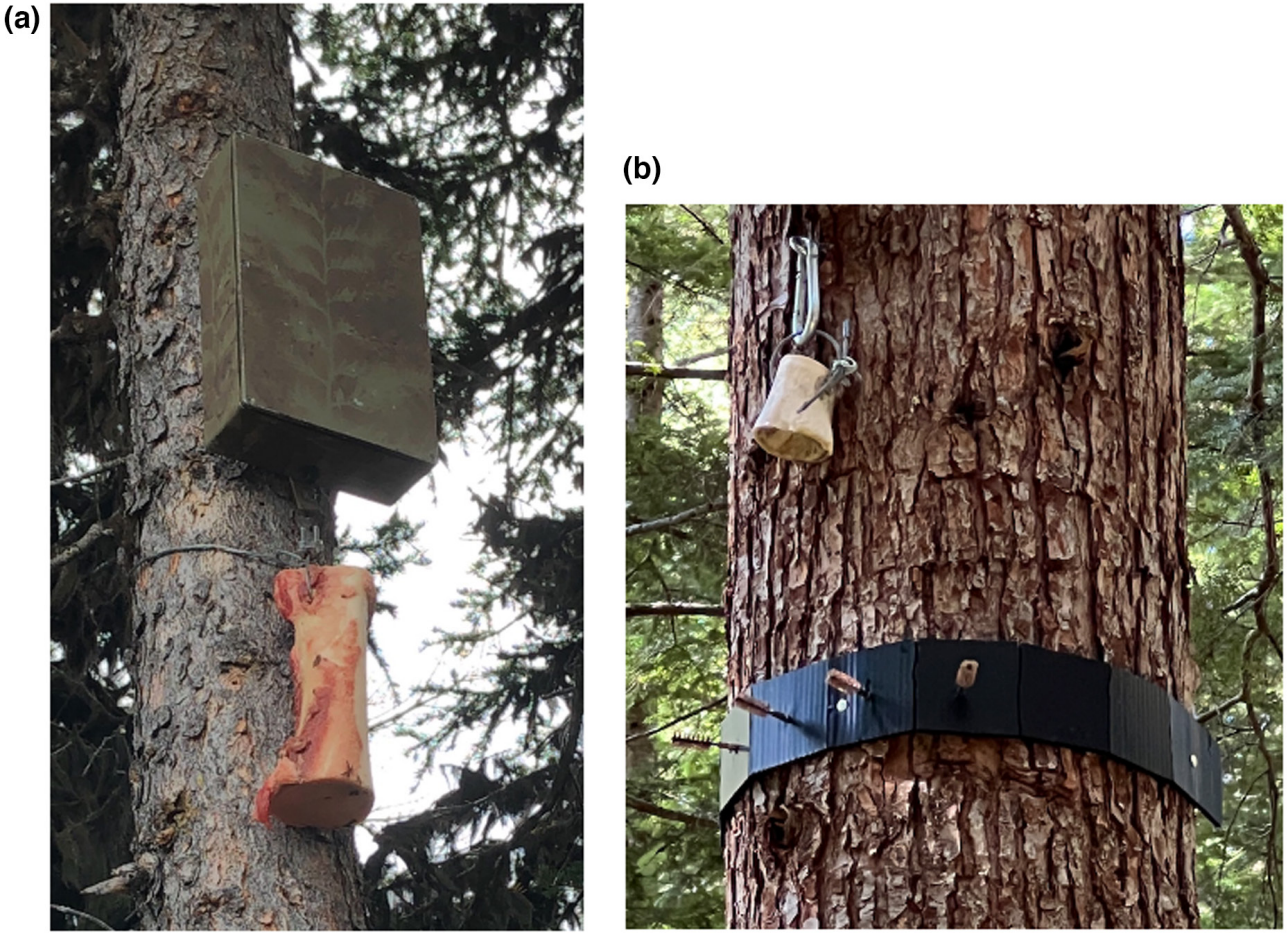
Two options for securing the bone under the scent dispenser: (a) a cable around the tree, and (b) an eyebolt (also note the hair‐snagging collar in “b”, which is optional).

At each survey station, we selected a tree measuring >30 cm in diameter at chest height, then bolted the dispenser to the bole at a height of 3–4 m from the ground. Dispensers were secured above the average mid‐winter snowpack depth to prevent accumulating snow from interfering with devices. To capture the dripping lure and provide a visual attractant, we secured a 20–25 cm section of a mostly meat‐free cow femur (*Bos taurus*) below the dispenser (Figure 3).

**FIGURE 4:**
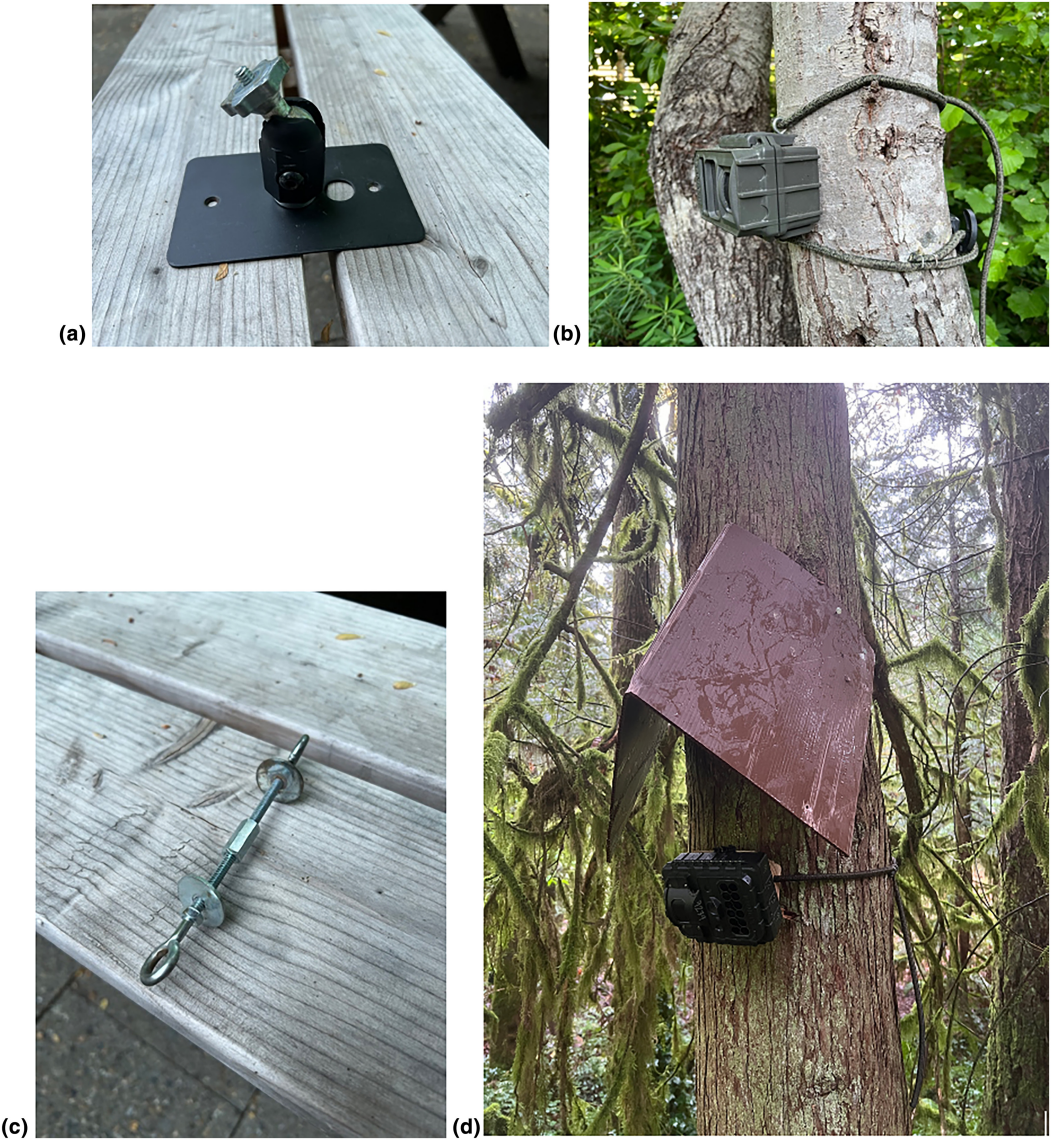
Two types of camera‐trap mounts that enable a 90° rotation of the camera trap: (a) a ball‐head, and (b) low‐cost eyelet hardware and elastic bands. Also, (c) detail of eyelet hardware, and (d) a camera trap mounted under a corrugated polypropylene roof.

On another tree of similar size, 4–5 m from the dispenser tree and within a clear line of sight, we mounted a Reconyx Ultrafire or Hyperfire CT at approximately the height of the dispenser. We rotated the CT 90° to create a “portrait” image orientation (Harley et al., 2014), enabling us to detect animals from the base of the target tree up to the dispenser, regardless of snow depth. CTs were mounted with various methods, including a lightweight eye‐and‐fastener system and a ball head mount (Figure 4). To collect as much photographic data as possible, we used 32 GB memory cards and programmed CTs to record five images per trigger event. We inserted a desiccant packet into the CT body to reduce condensation.

### 2.2 Study areas and survey design

We deployed CTs and automated scent dispensers in the North Cascades of Washington to detect wolverines, and in the Rocky Mountains of Idaho to detect fishers, as well as nontarget species in both regions. Surveys were conducted intensively in Washington (i.e., via a high density of stations in areas known to be used by wolverines; Aubry et al., 2023), and extensively in Idaho (i.e., at lower densities within a larger study area; Krohner et al., 2022).

#### 2.2.1 Washington

**FIGURE 5:**
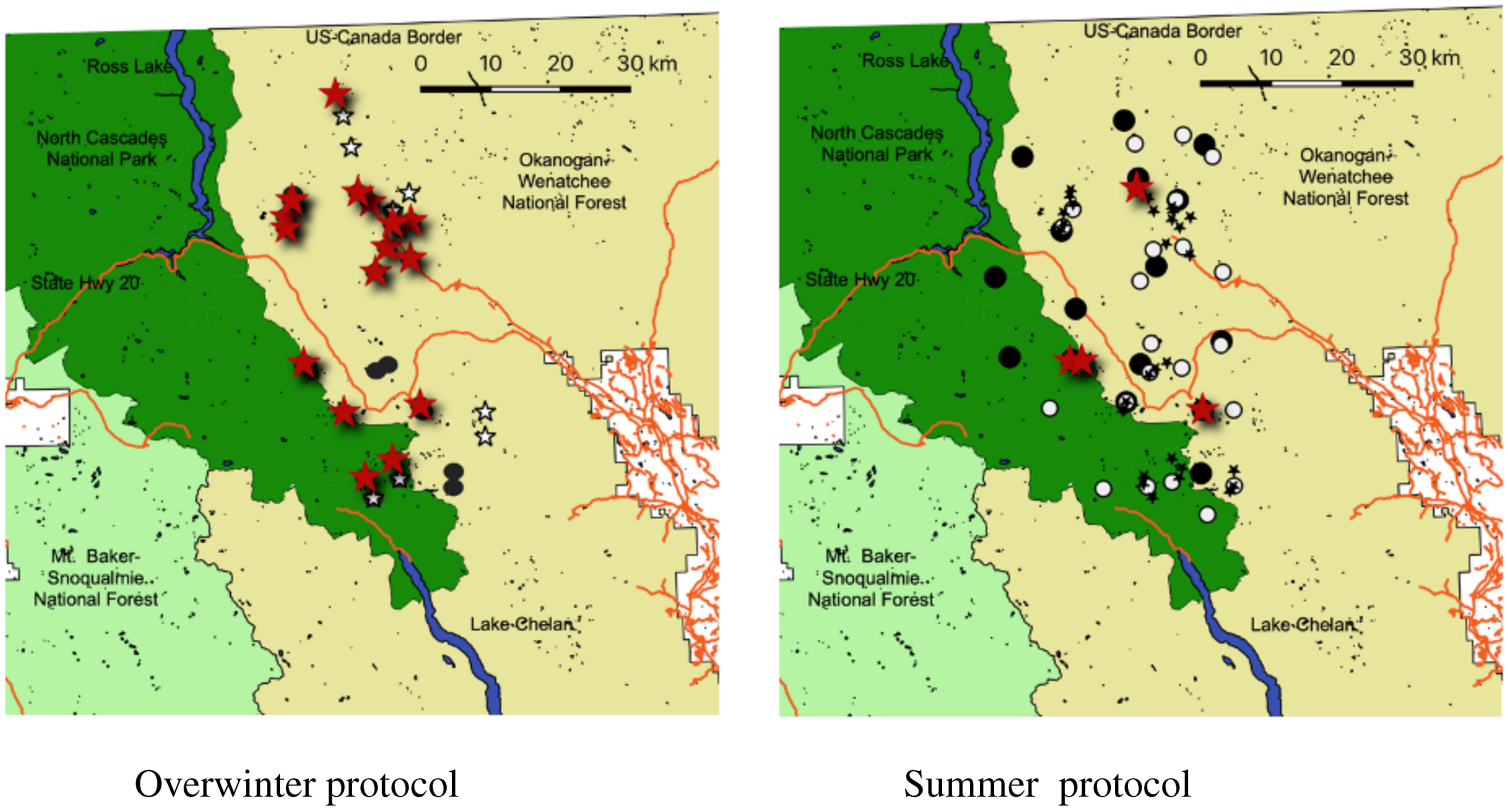
Left panel: locations of stations in Washington surveyed with the overwinter protocol during winters of 2015/16 (black symbols), and 2016/17 (star symbols), with resulting wolverine detections in all years (red stars). Note that some symbols overlap, when stations were surveyed in multiple years. Right panel: locations of stations surveyed with a standard summer protocol without scent dispensers during summers of 2013 (round black symbols), 2014 (white symbols), and 2015 (black star symbols), with resulting wolverine detections in all years (red stars). Note that some symbols overlap, where stations were surveyed in multiple years.

We implemented our protocol at 24 and 16 survey stations during the winters of 2015/16 and 2016/17, respectively (Figure 5). Survey stations were located within suitable wolverine habitat as defined by existing models (Copeland et al., 2010; Inman et al., 2013), and primarily within wolverine activity areas identified during a previous telemetry study (Aubry et al., 2023), in an effort to increase the chances that wolverines were present. We installed stations between July and October and returned the following summer. We filled the dispenser with ≤828 mL of liquid scent lure, which was sufficient to last ~515 days when programmed to release 3 mL of lure every other day. All stations were situated in mountainous locations at elevations ranging from 1524–2133 m. Snowfall averaged 3.5–4.5 m from October to April, year‐round rainfall ranged from 100–150 cm, and winters typically averaged a low temperature of −10°C from December to March (https://www.usclimatedata.com). Most sites could not have been safely or reliably accessed by field technicians during the winter.

For comparison purposes, we also present data previously collected by Long and MacKay in the North Cascades with another CT‐based protocol, employed only during summer and early fall (i.e., snow‐free periods) of 2013–2015 and in the same general region as our overwinter survey (Figure 5). This protocol entailed interim visits to stations by researchers for manual lure replenishment as opposed to dispersing scent via an automated scent dispenser. We used an existing run‐pole design (Magoun et al., 2011) and revisited stations once per month to deposit ~15 mL of olfactory lure (Caven's Gusto, Minnesota Trapline Products, Inc., Pennock, MN) on a 20–25 cm section of a mostly meat‐free cow femur suspended above the run‐pole. We deployed 15, 25, and 24 stations during 2013, 2014, and 2015, respectively. Stations were deployed during July and August and maintained through September or October.

#### 2.2.2 Idaho

We implemented our overwinter dispenser protocol at 39 stations across northern Idaho during the winter of 2018/19 as part of a multi‐state effort to estimate fisher occupancy (Krohner et al., 2022). Stations were deployed in October and November in modeled fisher habitat and removed the following spring or summer after snowmelt. Automated scent dispensers were programmed to release 3 mL of lure every day, with an anticipated depletion period of ~276 days. Elevations ranged from 1000–1850 m, and annual precipitation ranged from 1.1–1.7 m (https://www.usclimatedata.com), falling primarily as snow from November to April. All stations were deployed in mountainous locations that would have been very challenging to revisit safely in winter.

## 3 RESULTS

Results are summarized only for time periods during which survey devices were deemed fully operational.

### 3.1 Washington

**FIGURE 6:**
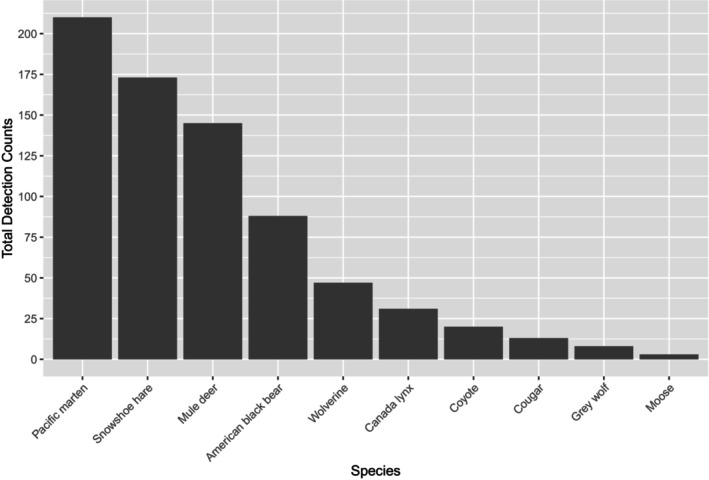
Total detection counts of various mammal species at 36 overwinter survey stations in Washington, 2015–2017. Detections were limited to those >24 hours apart for a particular species at a given station.

**FIGURE 7:**
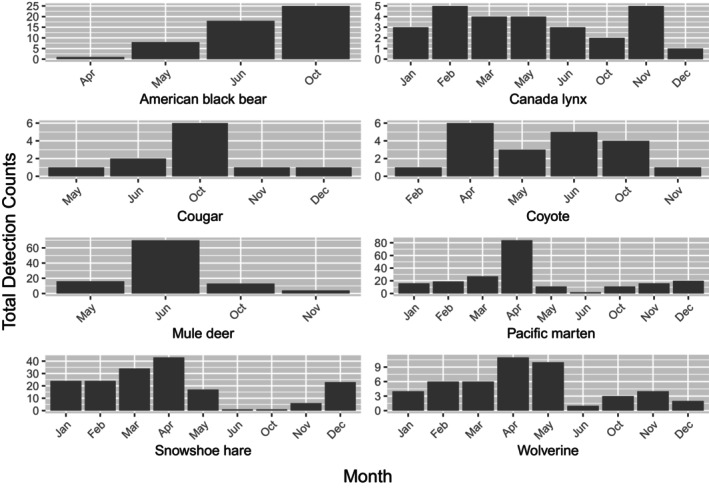
Total detection counts by month of various mammal species at 36 overwinter survey stations in Washington during winters 2015–2017. Detections were limited to those >24 hours apart for a particular species at a given survey station. Because the timing of station deployments and removals varied, we do not include summaries of detections in July–September.

**FIGURE 8:**
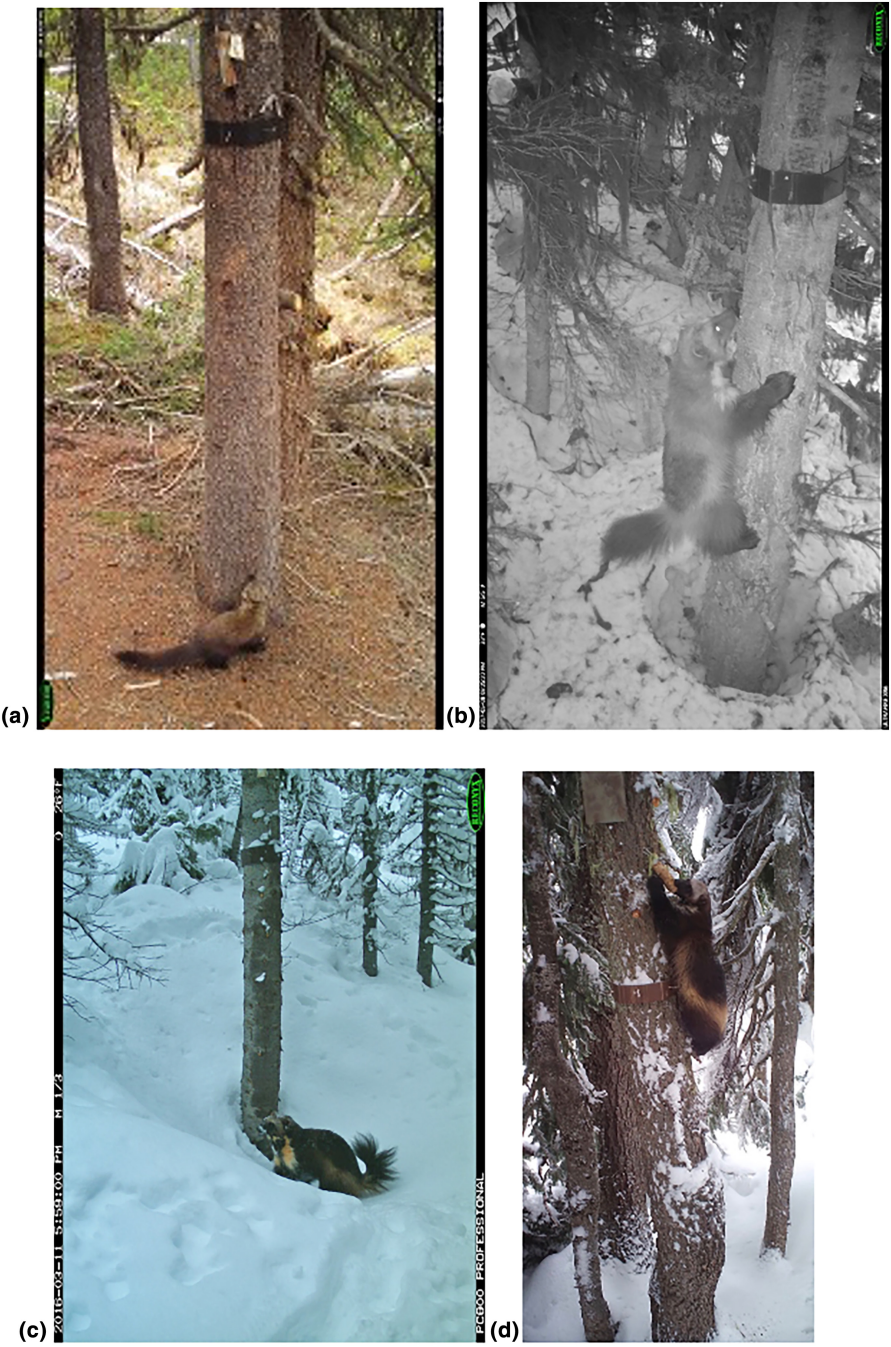
Camera trap images showing: (a) fisher at ground level during the non‐snow period, (b) wolverine on a tree bole in winter, and (c) wolverine on the deep snow surface in winter, and (d) wolverine chewing on the bone and rubbing against the hair‐snagging device.

**FIGURE 9:**
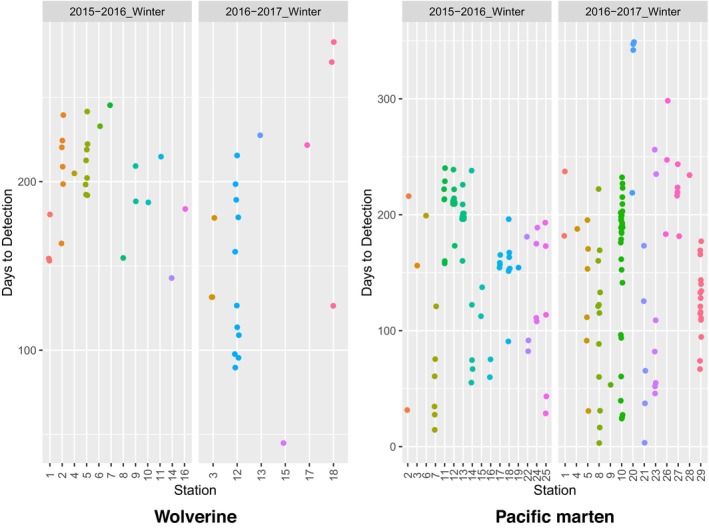
Days to detection for all detections of wolverines and Pacific martens at 18 and 29 overwinter survey stations, respectively, in Washington during winters 2015–2017. Detections were limited to those >24 h apart for a given survey station.

We determined that 37 of 40 stations were fully operational during our surveys, as indicated by functioning CTs and depleted lure when dispensers were removed. CTs at two stations were impeded by snow or disturbed by animals, and a scent dispenser malfunctioned at one other station due to loose wiring and excessive moisture. We subsequently modified the design of the dispenser to address these design issues (R. Long/J. Sauder, unpublished data). Despite frequent visits by American black bears (*Ursus americanus*), bears did not damage or dislodge any cameras or dispensers. CTs detected eight carnivore species, including wolverines, and >12 additional nontarget mammal species (Figure 6). Animals were detected throughout the year (Figure 7) and at all snow depths (Figure 8). We detected another extant mustelid, the Pacific marten (*Martes caurina*), at 29 stations (Figure 9). Fishers were considered extirpated from the North Cascades at the time of the surveys and were not detected.

We detected wolverines on 47 occasions, with a minimum of >24 h between detections, at 18 (48.6%) of the 37 stations (Figures 5 and 9). By comparison, CT/run‐pole surveys for wolverines conducted during summer/early fall, 2013–2015, yielded wolverine detections at only four of 80 stations (5.0%; Figure 5). Wolverine detection rates at overwinter CT/scent dispenser stations were four times higher than those at summer/early fall CT/run‐pole stations (Table 1).

**TABLE 1:** Comparison of the number of survey stations during a specific survey season, number of total survey days (days × stations) of effort, resulting wolverine detections (minimum >24 h apart for any given station), and resulting detection rate during three summer survey periods without scent dispensers and two overwinter survey periods using the overwinter (scent dispenser) protocol.

Season	Type of survey	# Survey stations	Total survey days	Wolverine detections	Detections per survey‐day
Summer 2013	No dispenser	16	803	1	0.001
Summer 2014	No dispenser	25	1707	1	0.001
Summer 2015	No dispenser	39	2822	2	0.001
Winter 2015–2016	Dispenser‐based	22	6727	27	0.004
Winter 2015–2016	Dispenser‐based	15	5443	20	0.004

**FIGURE 10:**
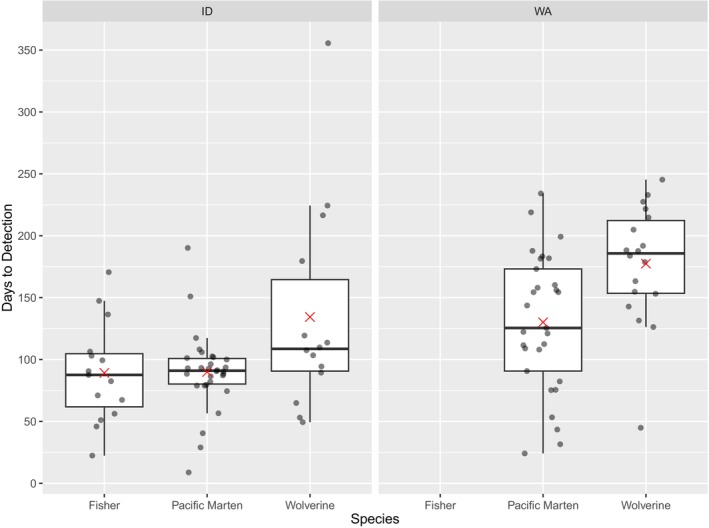
Days to first detection of target species. Each dot represents the first detection of a species at a given survey station. The boxes include the middle 50% of values in the dataset, and the bars within the boxes are the median. Red Xs are the means, and the whiskers cover up to the highest and lowest 25% of the dataset. Fishers were extirpated in the Washington Study area and were, therefore, undetected.

**FIGURE 11:**
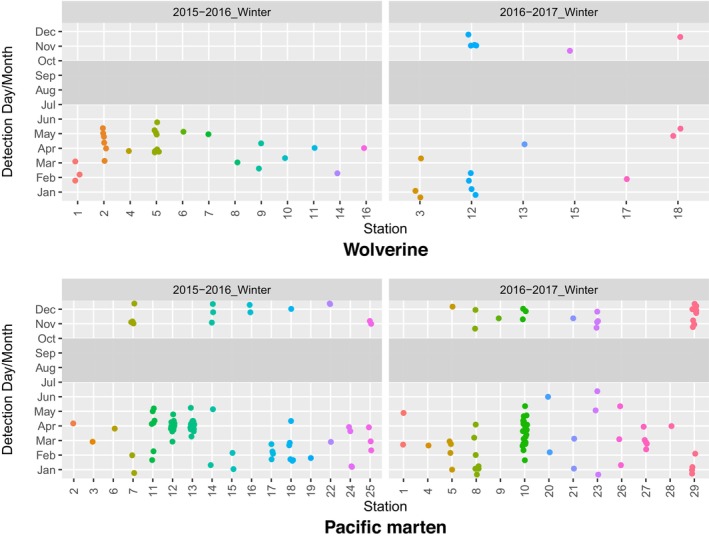
Day and month of detection for all detections of wolverines (top panel) and Pacific martens (bottom panel) at 18 and 29 overwinter survey stations, respectively, in Washington, 2015–2017. Detections were limited to those >24 h apart at a given survey station. July–September detections are not shown, as deployments varied in terms of survey effort during those months.

Time to first detection at overwinter stations averaged 177 days for wolverines and 130 days for Pacific martens (Figure 10), and detections of both species occurred throughout the winter (Figure 11). Further, 77% of wolverine visits occurred >5 months after station deployment, indicating that wolverines were attracted to stations with automated scent dispensers long after station deployment.

### 3.2 Idaho

**FIGURE 12:**
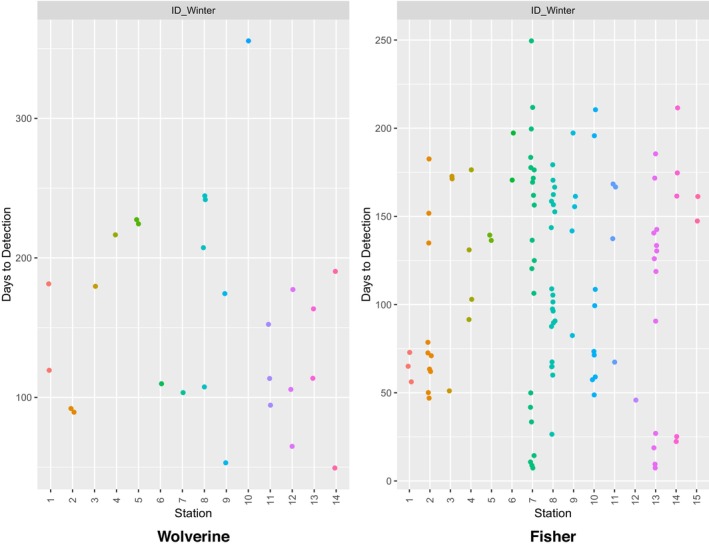
Days to detection for all detections of wolverines and fishers at 14 and 15 overwinter survey stations, respectively, in Idaho during winters 2015–2017. Detections were limited to those >24 h apart for a given survey station.

**FIGURE 13:**
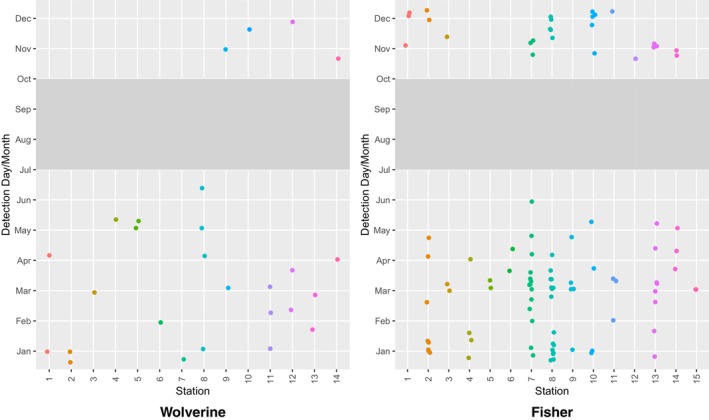
Day and month of detection for all detections of wolverines and fishers at 14 and 15 overwinter survey stations in Idaho. Detections were limited to those >24 h apart for at a given survey station. July–September detections are not shown, as deployments varied in terms of survey effort during those months.

Of 39 CT stations deployed with automated scent dispensers, 37 remained fully operational throughout the survey period (two dispensers malfunctioned due to unknown causes). There were no CT malfunctions, and nor were any CTs disturbed by animals (note that, in this study area, all CTs were protected within metal security boxes to prevent damage by bears). Fishers were detected at 15 of 37 stations (41%), wolverines at 14 of 37 stations (41%), and martens (*Martes americana* or *Martes caurina*) at 30 of 37 stations (81%). Overall, we detected 11 carnivore species and seven other mammal species. As in Washington, animals were detected throughout the survey period (Figures 12 and 13). Time to first detection averaged 134 days for wolverines, 89 days for fishers, and 90 days for martens (Figure 10).

## 4 DISCUSSION

Our results show that a vertically positioned CT, paired with an automated scent dispenser, can effectively and repeatedly detect wolverines, fishers, and other carnivores for at least a year, including during winters characterized by very cold temperatures and deep snowpack. Detection patterns for all species largely conformed to our expectations. For example, black bears were detected only in April through October (i.e., the nondenning months), and wolverines and martens mostly in winter. Coyotes (*Canis latrans*), Canada lynx (*Lynx canadensis*), and cougars (*Puma concolor*) were detected year‐round (Figure 7), and mule deer (*Odocoileus hemionus*) primarily during summer and fall, when our high‐elevation stations were presumably more accessible to these animals.

Previous studies found no difference in detection probabilities for wolverines (Lukacs et al., 2020) or fishers (Krohner et al., 2022, using portions of the same data analyzed here) at CT stations paired with automated scent dispensers per our protocol versus those regularly replenished with meat baits and scent lures. These findings, in combination with cost savings and reduced risks associated with not having to visit survey stations in winter, led to the widespread application of our survey protocol for wolverine surveys conducted during winter 2021/22 in Montana, Idaho, Wyoming, Colorado, Utah, Oregon, and Washington, and for fisher surveys conducted during winter 2023/24 across Idaho and Washington (C. Mosby [Idaho Fish and Game], J. Lewis [Washington Fish and Wildlife], pers. comm.). Our protocol was also used in Yellowstone National Park during a multi‐state wolverine survey (Lukacs et al., 2020), where typical meat baits were not allowed due to concerns about potential interactions between people and grizzly bears (*Ursus arctos*), as well as other carnivores, at baited stations.

As a hypothetical example of cost savings, a traditional CT survey conducted for 4 months, assuming at least one revisit per month to replenish baits and lures, would require twice the number of person‐days compared with a survey of similar length using automated scent dispensers. The cost of the scent dispenser at the time of this study was ~$300 USD, and lure expenses per deployment were <$5 USD. Given the expense of deploying a single field crew visit to rebait/relure CTs in relatively accessible areas, we believe our protocol will yield a large cost savings for many if not most CT‐based carnivore surveys carried out over multiple months and across remote, expansive terrain. Actual costs are highly dependent upon factors specific to a survey area, such as personnel availability and regional salary rates, transportation needs, permit limitations, and distance to and accessibility of CT stations.

While it is common for researchers to maintain remote CT stations in winter using motorized or nonmotorized winter transportation (e.g., all‐wheel‐drive vehicles, snowmobiles, helicopters, skis, snowshoes), options vary dramatically by region. In our Washington study area, for example, winter access to survey stations is limited by few roads and steep avalanche terrain, and research permits for sites with official Wilderness designation generally preclude helicopter activity. Because our protocol allows CT stations to be deployed in summer and removed a year later, stations can typically be accessed by hiking trail or forest road during snow‐free seasons—minimizing cost, training requirements, and risks associated with winter field efforts. These factors may increase the pool of people capable of deploying stations and can potentially help researchers leverage field collaborations with agencies, Tribes, and NGOs to expand survey capacity and extent. Even in places where winter access is feasible, our protocol eliminates the need for labor‐intensive revisits.

Because the scent dispenser precludes the need for meat baits, it also eliminates the potential for the spread of transmissible diseases—for example, chronic wasting disease (CWD). CWD is a growing concern in a number of US states and Canadian provinces and has resulted in regulations and guidelines designed to slow or stop its spread (e.g., https://cwd‐info.org/carcass‐transportation‐regulations‐in‐the‐united‐states‐and‐canada/). Indeed, some biologists working in states where CWD occurs, or adjacent to such states, report reduced ability to acquire and transport meat baits for use in CT research (J. Sauder, unpublished data).

The scent dispenser obviates the “attractiveness decay” factor that may limit the effectiveness of unrefreshed baits and lures over time (Buyaskas et al., 2020)—an important consideration for wolverine surveys, given that the time to first detection can be lengthy. Even when baits are periodically refreshed, variations in bait size, condition, and type, as well as environmental factors such as precipitation, temperature, and the presence of bait scavengers, can lead to variation in detection probability (e.g., Zielinski et al., 2015). The scent dispenser also minimizes human disturbance and scent at CT stations—factors that could contribute to site avoidance by target species. One study reported a decrease in the short‐term detectability of predators and prey at CT stations due to revisits by field personnel to refresh attractants (Mills et al., 2019), and wolverines in particular have been shown to be sensitive to human disturbance during winter denning periods (Heinemeyer et al., 2019).

The scent dispenser's long operating period also affords flexibility for data analyses. Analysts can choose to parse lengthy survey periods to fit research needs and adhere to assumptions of selected statistical methods. For example, if occupancy patterns during the wolverine denning period (i.e., typically Feb–May) were of primary interest, camera data could be extracted from the larger dataset and analyzed for that period.

Our protocol clearly has the potential to be effective for surveying carnivores beyond mustelids, as demonstrated by the detection of nontarget species in this study (Figure 6). The scent dispenser is highly adaptable, enabling researchers to program the amount of scent lure to be released (i.e., 0.5–15.0 mL) and at what frequency (i.e., every 6 h–30 days), and to disburse species‐specific lures to meet project objectives.

As with any survey protocol, ours does have its limitations. Buyaskas et al. (2020) found that lures alone yield lower detection rates for some carnivores (e.g., mustelids) than when coupled with bait. These researchers note, however, that lure degradation may be a factor here—a concern that, as mentioned above, is addressed by the automated scent dispenser. Conversely, a consistent olfactory source, such as that provided by the scent dispenser, may result in animal habituation, potentially decreasing repeat detections of individuals since they are not being rewarded. Repeat species‐level detections are necessary for some analytical methods (e.g., occupancy estimation [MacKenzie et al., 2002]), while others, such as spatial capture–recapture analyses (Efford, 2004), require the ability to track repeat visits by individuals. Our study was not set up to identify individuals, as we did not deploy run‐poles (see Magoun et al., 2011) during overwinter surveys, but we did observe repeat species‐level visits by wolverines, fishers, and martens across both study areas (Figures 8, 10, 11, 12). Our protocol could potentially be combined with a run‐pole to provide individual ID data.

Field personnel should take care to minimize vegetation that could trigger or block the CT's view, including branches that might droop into the viewshed under heavy snow conditions. The vertically oriented CT should be aimed such that the bole of the target tree is precisely aligned with the sensors, which vary by CT make and model. Because orienting the CT vertically reduces its horizontal detection zone at ground level, animals who visit the CT station but do not approach the base of the target tree may be undetected. If this is a concern for a particular target species, we recommend securing a second CT to a different tree to record photos from another angle—also providing insurance in case the primary CT malfunctions.

Hair‐snagging devices can be deployed in conjunction with this protocol to obtain DNA samples (see Figures 1 and 2 for a sample design). However, if hair samples are exposed to UV light for prolonged periods of time, DNA samples may be damaged and unsuitable for genetic testing. Because hair samples could be exposed to ambient conditions for up to a year with our protocol, hair‐snagging devices may be of limited use for collecting supplemental data unless hairs are deposited not long before researchers return to the station. We encourage researchers who seek to combine our protocol with hair‐snagging devices to explore new methods for shielding hair samples from UV light and precipitation in the field in order to maximize DNA viability.

Perhaps the most significant limitation of our protocol is that CTs or scent dispensers that are rendered inoperable by animals, weather, or equipment failure may be left unchecked for up to a year, resulting in many months of lost data. Although we did not experience issues with black bears disturbing camera stations, we do not know if grizzlies would present a greater challenge in this regard. The authors successfully tested the outer housings of two scent dispenser devices for grizzly bear durability by filling them with odorous materials and food and presenting them to two 300+ kg adult grizzlies under human care.

We have demonstrated that our CT/scent dispenser protocol can generate detection data for wolverines, fishers, and several other mammalian species through harsh, snowy winters and over the course of a year. This setup requires no revisits by researchers during long deployments and is largely resistant to damage by wildlife.

## AUTHOR CONTRIBUTIONS

**Robert A. Long:** Conceptualization (lead); formal analysis (lead); funding acquisition (lead); investigation (lead); methodology (lead); project administration (lead); resources (lead); supervision (lead); writing – original draft (lead); writing – review and editing (lead). **Paula MacKay:** Conceptualization (lead); funding acquisition (lead); investigation (lead); methodology (lead); project administration (lead); resources (lead); supervision (lead); writing – original draft (lead); writing – review and editing (lead). **Joel D. Sauder:** Conceptualization (lead); funding acquisition (lead); investigation (lead); methodology (lead); project administration (lead); resources (lead); supervision (lead); writing – review and editing (equal). **Mike Sinclair:** Methodology (equal); resources (equal); writing – review and editing (equal). **Keith B. Aubry:** Conceptualization (supporting); funding acquisition (supporting); writing – review and editing (equal). **Catherine M. Raley:** Conceptualization (supporting); funding acquisition (supporting); investigation (supporting); writing – review and editing (equal).

## CONFLICT OF INTEREST STATEMENT

The authors have no conflict of interest to declare.

## Data Availability

The raw data used in this paper are available on Zenodo at https://doi.org/10.5281/zenodo.10882394.

## References

[ece311290-bib-0001] Anderson, A. K. , Waller, J. S. , & Thornton, D. H. (2023). Canada lynx occupancy and density in Glacier National Park. Journal of Wildlife Management, 87, e22383. 10.1002/jwmg.22383

[ece311290-bib-0002] Aubry, K. B. , Raley, C. M. , Shirk, A. J. , McKelvey, K. S. , & Copeland, J. P. (2023). Climatic conditions limit wolverine distribution in the Cascade Range of southwestern North America. Canadian Journal of Zoology, 101, 95–113.

[ece311290-bib-0003] Barcelos, D. C. , Alvarenga, G. C. , Gräbin, D. M. , Baccaro, F. , & Ramalho, E. E. (2023). Divergent effects of lure on multi‐species camera‐trap detections and quality of photos. Journal for Nature Conservation, 71, 126317. 10.1016/j.jnc.2022.126317

[ece311290-bib-0004] Burton, A. C. , Beirne, C. , Sun, C. , Granados, A. , Procko, M. , Chen, C. , Fennell, M. , Constantinou, A. , Colton, C. , Tjaden‐McClement, K. , Fisher, J. T. , & Burgar, J. (2022). Behavioral “bycatch” from camera trap surveys yields insights on prey responses to human‐mediated predation risk. Ecology and Evolution, 12(7). Portico. 10.1002/ece3.9108 PMC9288887 35866017

[ece311290-bib-0005] Burton, A. C. , Neilson, E. , Moreira, D. , Ladle, A. , Steenweg, R. , Fisher, J. T. , Bayne, E. , & Boutin, S. (2015). Wildlife camera trapping: A review and recommendations for linking surveys to ecological processes. Journal of Applied Ecology, 52, 675–685. 10.1111/1365-2664.12432

[ece311290-bib-0006] Buyaskas, M. , Evans, B. E. , & Mortelliti, A. (2020). Assessing the effectiveness of attractants to increase camera trap detections of North American mammals. Mammalian Biology, 100, 91–100. 10.1007/s42991-020-00011-3

[ece311290-bib-0029] Caravaggi, A. , Burton, A. C. , Clark, D. A. , Fisher, J. T. , Grass, A. , Green, S. , Hobaiter, C. , Hofmeester, T. R. , Kalan, A. K. , Rabaiotti, D. , & Rivet, D. (2020). A review of factors to consider when using camera traps to study animal behavior to inform wildlife ecology and conservation. Conservation Science and Practice, 2(8). Portico. 10.1111/csp2.239

[ece311290-bib-0007] Copeland, J. P. , McKelvey, K. S. , Aubry, K. B. , Landa, A. , Persson, J. , Inman, R. M. , Krebs, J. , Lofroth, E. , Golden, H. , Squires, J. R. , Magoun, A. , Schwartz, M. K. , Wilmot, J. , Copeland, C. L. , Yates, R. E. , Kojola, I. , & May, R. (2010). The bioclimatic envelope of the wolverine (*Gulo gulo*): Do climatic constraints limit its geographic distribution? Canadian Journal of Zoology, 88, 233–246.

[ece311290-bib-0008] Efford, M. G. (2004). Density estimation in live‐trapping studies. Oikos, 106, 598–610.

[ece311290-bib-0009] Farris, Z. J. , Kelly, M. J. , Karpanty, S. , Murphy, A. , Ratelolahy, F. , Andrianjakarivelo, V. , & Holmes, C. (2017). The times they are a changin': Multi‐year surveys reveal exotics replace native carnivores at a Madagascar rainforest site. Biological Conservation, 206, 320–328. 10.1016/j.biocon.2016.10.025

[ece311290-bib-0010] Green, A. M. , Chynoweth, M. W. , & Şekercioğlu, Ç. H. (2020). Spatially explicit capture‐recapture through camera trapping: A review of benchmark analyses for wildlife density estimation. Frontiers in Ecology and Evolution, 8, e563477. 10.3389/fevo.2020.563477

[ece311290-bib-0011] Harley, D. K. , Holland, G. J. , Hradsky, B. A. K. , & Antrobus, J. S. (2014). The use of camera traps to detect arboreal mammals: Lessons from targeted surveys for the cryptic Leadbeater's Possum (Gymnobelideus leadbeateri). In P. Meek & P. Fleming (Eds.), Camera trapping: Wildlife management and research (pp. 233–243). Csiro Publishing.

[ece311290-bib-0012] Heinemeyer, K. , Squires, J. , Hebblewhite, M. , O'Keefe, J. J. , Holbrook, J. D. , & Copeland, J. (2019). Wolverines in winter: Indirect habitat loss and functional responses to backcountry recreation. Ecosphere, 10, e02611.

[ece311290-bib-0013] Holinda, D. , Burgar, J. M. , & Burton, A. C. (2020). Effects of scent lure on camera trap detections vary across mammalian predator and prey species. PLoS One, 15, e0229055. 10.1371/journal.pone.0229055 32396558 PMC7217433

[ece311290-bib-0014] Iannarilli, F. , Erb, J. , Arnold, T. W. , & Fieberg, J. R. (2021). Evaluating species‐specific responses to camera‐trap survey designs. Wildlife Biology, 1, wlb.00726. 10.2981/wlb.00726

[ece311290-bib-0015] Inman, R. M. , Brock, B. L. , Inman, K. H. , Sartorius, S. S. , Abera, B. C. , Giddings, B. , Cain, S. L. , Orme, M. L. , Fredricki, J. A. , Oakleaf, B. J. , Altg, K. L. , Odell, E. , & Chapron, G. (2013). Developing priorities for metapopulation conservation at the landscape scale: Wolverines in the western United States. Biological Conservation, 166, 276–286. 10.1016/j.biocon.2013.07.010

[ece311290-bib-0016] Kays, R. W. , & Slauson, K. M. (2008). Remote cameras. In R. A. Long , P. MacKay , W. J. Zielinski , & J. C. Ray (Eds.), Noninvasive survey methods for carnivores (pp. 110–140). Island Press.

[ece311290-bib-0017] Kellner, K. F. , Parsons, A. W. , Kays, R. , Millspaugh, J. J. , & Rota, C. T. (2022). A two‐species occupancy model with a continuous‐time detection process reveals spatial and temporal interactions. Journal of Agricultural, Biological and Environmental Statistics, 27, 321–338. 10.1007/s13253-021-00482-y

[ece311290-bib-0018] Krohner, J. M. , Lukacs, P. M. , Inman, R. , Sauder, J. D. , Gude, J. A. , Mosby, C. , Coltrane, J. A. , Mowry, R. A. , & Millspaugh, J. J. (2022). Finding fishers: Determining fisher occupancy in the Northern Rocky Mountains. The Journal of Wildlife Management, 86, 1–20. 10.1002/jwmg.22162

[ece311290-bib-0019] Lukacs, P. , Evans Mack, D. , Inman, R. , Gude, J. , Ivan, J. , Lanka, R. , Lewis, J. , Long, R. , Walker, Z. , Courville, S. , Kahn, R. , Schwartz, M. , Torbit, S. , Waller, J. , & Carroll, K. (2020). Wolverine occupancy, spatial distribution, and monitoring design. Journal of Wildlife Management, 84, 841–851. 10.1002/jwmg.21856

[ece311290-bib-0020] MacKenzie, D. I. , Nichols, J. D. , Lachman, G. B. , Droege, S. , Royle, J. A. , & Langtimm, C. A. (2002). Estimating site occupancy rates when detection probabilities are less than one. Ecology, 83, 2248–2255.

[ece311290-bib-0021] Magoun, A. J. , Long, C. D. , Schwartz, M. K. , Pilgrim, K. L. , Lowell, R. E. , & Valkenburg, P. (2011). Integrating motion‐detection cameras and hair snags for wolverine identification. Journal of Wildlife Management, 75, 731–739.

[ece311290-bib-0022] Mills, D. , Fattebert, J. , Hunter, L. , & Slotow, R. (2019). Maximizing camera trap data: Using attractants to improve detection of elusive species in multi‐species surveys. PLoS One, 14, e0216447. 10.1371/journal.pone.0216447 31141506 PMC6541258

[ece311290-bib-0023] Murphy, A. , Diefenbach, D. R. , Ternent, M. , Lovallo, M. , & Miller, D. (2021). Threading the needle: How humans influence predator–prey spatiotemporal interactions in a multiple‐predator system. Journal of Animal Ecology, 90, 2377–2390. 10.1111/1365-2656.13548 34048031

[ece311290-bib-0024] Rich, M. , Thompson, C. , Prange, S. , & Popescu, V. D. (2018). Relative importance of habitat characteristics and interspecific relations in determining terrestrial carnivore occurrence. Frontiers in Ecology and Evolution, 6, 78. 10.3389/fevo.2018.00078

[ece311290-bib-0025] Rowcliffe, J. M. , Field, J. , Turvey, S. T. , & Carbone, C. (2008). Estimating animal density using camera traps without the need for individual recognition. Journal of Applied Ecology, 45, 1228–1236.

[ece311290-bib-0026] Saunders, S. E. , Bartelt‐Hunt, S. L. , & Bartz, J. C. (2012). Occurrence, transmission, and zoonotic potential of chronic wasting disease. Emerging Infectious Diseases, 18, 369–376. 10.3201/eid1803.110685 22377159 PMC3309570

[ece311290-bib-0027] Zielinski, W. J. , Moriarty, K. M. , Baldwin, J. , Kirk, T. A. , Slauson, K. M. , Rustigian‐Romsos, H. L. , & Spencer, W. D. (2015). Effects of season on occupancy and implications for habitat modeling: The Pacific marten *Martes caurina* . Wildlife Biology, 21, 56–67. 10.2981/wlb.00077

[ece311290-bib-0028] Zielinski, W. J. , Moriarty, K. M. , Kirk, T. A. , & Slauson, K. M. (2011). Understanding seasonal variation in detection of martens using radio‐marked individuals. Final report to the Lassen National Forest, 7 September. USDA Pacific Southwest Research Station, Arcata, California, & Oregon State University, Department of Fisheries and Wildlife, Corvallis, Oregon.

